# Peanut-Shell Biochar and Biogas Slurry Improve Soil Properties in the North China Plain: A Four-Year Field Study

**DOI:** 10.1038/s41598-018-31942-0

**Published:** 2018-09-13

**Authors:** Zhenjie Du, Yatao Xiao, Xuebin Qi, Yuan Liu, Xiangyang Fan, Zhongyang Li

**Affiliations:** 1 Farmland Irrigation Research Institute, Chinese Academy of Agricultural Sciences, Xinxiang, 453003 China; 2 Agriculture Water and Soil Environmental Field Science Research Station of Xinxiang City of Henan Province of CAAS, Xinxiang, 453003 China; 3 College of Resources and Environmental Sciences, Nanjing Agricultural University, Nanjing, 210095 China

## Abstract

Biochar and biogas slurry have been proved to improve the quality of some soil types, but the long-term effects on fluvo-aquic soil are not fully understood. This study aimed to compare the continuity effects of peanut-shell biochar and biogas slurry on the physicochemical properties, microbial population size, and enzyme activities of fluvo-aquic soil. We conducted a four-year field experiment of winter wheat-summer maize rotation in the North China Plain. Along with equal nitrogen inputs, three treatments were applied—conventional fertilizers, peanut-shell biochar, and hoggery biogas slurry—after which various soil quality indicators were compared. Compared with those of control, both biochar and biogas slurry increased the soil total nitrogen and organic matter content, and improved soil aggregation, microbial biomass, and actinomycetes. Biogas slurry decreased soil pH and improved urease and protease activities. With biochar and biogas slurry treatments, wheat yield increased by 8.46% and 23.47%, and maize yield by 18% and 15.46%, respectively. Biogas slurry increased the content of crude protein and starch in the grains. Both biogas slurry and peanut-shell biochar improved fluvo-aquic soil nutrient content, water-stable macroaggregates, and microbial population, which might be related to their high nutrient content, large specific surface area, adsorption capacity, and functional groups. Biogas slurry generally exhibited stronger effects than biochar probably because of its richness in nutrients and bioactive substances.

## Introduction

In China, excessive fertilizer application has been a common practice over the past decades to achieve high crop productivity, which has resulted in the degradation of soil and contamination of both surface and groundwater^1^. Rotation of winter wheat and summer maize (or peanut) is the main cultivation method in the North China Plain, with wheat sown in October and harvested in June, and maize (peanut) sown in June (May) and harvested in October. In the short span between maize (peanut) harvest and wheat sowing, farmers have to remove the maize (peanut) straw in time for wheat sowing. Over the past decades, farmers have taken the easy option of burning the straws on site, leading to serious air pollution^2^. Similarly, the livestock industry in China has substantially grown over the past two decades, generating enormous amounts of solid and liquid wastes. With the increased public awareness of environmental issues and food safety, the use of livestock wastes and crop straws has received attention over the past decades^3–5^. For livestock wastes, the common method employed is the application of slurry generated from their treatments to improve soil fertility. Crop straws can be converted into biochar, which is applied to soil to promote carbon sequestration and soil amendment^5,6^.

Biochar is a type of charcoal produced by heating crop waste and other biomass in a simple kiln designed to limit the presence of oxygen. Biogas slurry is the residue of crops straw, and animal and human excreta after anaerobic fermentation. Most studies on biochar have focused on its physicochemical characteristics^7^, conversion, behaviour in different environments^8–10^, and its effects on crop yield^11^, greenhouse-gas emission^12^, and biogeochemical cycling^13^. Studies on the application of biogas slurry to agricultural lands has mainly focused on its effect on crop growth and soil quality^5,14^, soil nutrients and greenhouse-gas emissions^15,16^, and soil biochemical processes^17,18^. Most studies have shown that either biochar or biogas slurry can increase soil fertility and structure, and consequently, crop productivity.

Most studies on the effects of biogas slurry and biochar on soil fertility and functions have been based on short-term experiments, lasting just one year or even one season. However, field studies on the long-term effects of biochar and slurry application are limited. Major *et al*.^19^ showed that biochar can increase maize grain yield, nutrient uptake, and soil (Savanna Oxisol) pH based on the results of a four-year field study in Colombia. They attributed yield-improving effect to higher available Ca and Mg content. In China, studies on amending soils with organic matter (OM) have focused on soil physicochemical properties in arid and semi-arid areas in the northwest region of the country and changes in the structure of red soils in the south^20,21^. Soils in the North China Plain are predominantly fluvo-aquic soils (Chinese Soil Taxonomy, entisols in US Soil Taxonomy), which have low fertility due to limited humus accumulation and poor soil structure (heavy-textured soil or heavy sandy soil). As one of the most important agricultural production regions, the North China Plain accounts for approximately one fifth of the cultivated area of China, with an area of 350,000 km^2^ and approximately 18 million ha of agricultural land. Therefore, there is a large spatial variability in soil properties among different regions in the North China Plain. In general, the soil pH ranges from 8.16 to 8.70 (alkaline), organic matter is 4.72–19.62 g/kg, bulk density is 1.33–1.43 g/cm^3^, total nitrogen content is 0.44–0.89 g/kg, total phosphorus content is 0.50–1.03 g/kg, total potassium content is 17.60–20.31 g/kg^5,22,23^. Studies on the effects, especially the long term effects, of biochar and biogas slurry on structure, fertility, and other properties of fluvo-aquic soils in Northern China are limited^5^. In addition, due to irrational fertilization and over-extraction, the structure and fertility of fluvo-aquic soils in Northern China have been deteriorating, posing an obstacle to sustainable agriculture development^24^.

We assumed that (1) biochar has positive effects on soil nutrients, microbe population, and enzyme activities because of its unique features-BET (Brunauer-Emmett-Teller) surface area, low bulk density, and porous structure^25,26^ and (2) biogas slurry can potentially improve soil physicochemical properties because of its unique characteristics—high humic acid content, functional groups of hydrophilic colloid, and chemical bonds^17^. To validate our hypothesis, we conducted a four-year field experiment with consecutive wheat-maize rotations in the fluvo-aquic soils from the North China Plain, involving three treatments: conventional fertilizers, hoggery biogas slurry, and peanut-shell biochar (all the treatments included fertilization with equal amount of nitrogen). In this study, we observed soil nutrition content, soil aggregates, microbe abundance, enzyme activities, crop yield, and other defining characteristics under different treatment conditions. We aimed to elucidate the reasons for the differences in soil function parameters in order to understand the response mechanisms of soil properties to hoggery biogas slurry and biochar treatments. These results could provide scientific guidance for soil sustainable productivity and environmental security by the efficient utilization of biomass waste resources.

## Results

### Chemical properties of the top soil

The chemical properties of the top soil under different treatment conditions are shown in Table 2. The effect of soil amendment with biochar and slurry on soil pH is shown in Tables 1 and 2. The soil pH with biogas slurry treatment (BS) decreased during the first two years (2011–2012), while it remained nearly unchanged with the biochar treatment (BC). The soil total nitrogen (TN) and total phosphorus (TP) content increased with the BS treatment, whereas, the content organic matter (OM) increased during the first year, but did not change thereafter. In contrast, with BC treatment, the content of TN and OM increased significantly in 2011, and thereafter, declined continuously; however, their overall levels at the end of the experiment were still higher than those before the experiment. The soil OM content decreased in 2012 and then remained constant in the control (CK) plots. Compared with the background values shown in Table 1, with the BS treatment, the soil TN and TP content increased in 2013–2014 and the soil OM content increased in 2011–2014. With the BC treatment, the soil TN and TP content increased in 2011–2014 and the soil OM content increased in 2011–2013. Compared with that of the CK, the pH of soil subjected to BS treatment decreased 2011–2013.

**Table 1:** Physical and chemical properties of the soil before the experiment.

Depth (cm)	pH	Bulk density (g/cm^3^)	Total porosity (%)	Soil mechanical composition (g/kg)			Soil texture	Total N (g/kg)	Total P (g/kg)	Organic matter (g/kg)
				Clay particles	Silt particles	Sand particles				
0–20	8.16	1.39	47.55	164.13	465.93	369.94	silty loam	0.89	0.76	19.62
20–40	8.34	1.33	49.81	186.31	367.24	446.45	clay loam	0.88	0.83	9.63
40–60	8.50	1.49	43.77	283.14	458.19	258.67	silty clay loam	0.55	0.75	4.13

**Table 2:** Effect of different treatments on the physical and chemical properties of the 0–20 cm soil layer after the harvest of winter wheat in 2011–2014a.

Year	Treatment	Soil physical and chemical properties					
		Bulk density (g/cm^3^)	Total porosity (%)	pH	Total N (g/kg)	Total P (g/kg)	Organic matter (g/kg)
2011	CK	1.41a	46.38e	8.03b	0.78g	0.72cd	19.83c
	BS	1.40ab	46.83e	7.82c	0.93ef	0.79b	22.09a
	BC	1.32d	50.02b	8.36a	1.41a	0.82b	22.62a
2012	CK	1.38b	47.92d	8.30a	0.85fg	0.76c	19.11d
	BS	1.37b	48.23d	7.56c	1.32b	0.86ab	22.32a
	BC	1.35c	49.06c	8.24a	1.24c	0.88a	21.14b
2013	CK	1.38b	47.92d	8.23ab	0.97c	0.81b	18.91d
	BS	1.34cd	49.43bc	7.46d	1.29bc	0.84b	21.93a
	BC	1.29e	51.32a	8.39a	1.13d	0.91a	20.44bc
2014	CK	1.36bc	48.68cd	8.30a	1.02e	0.76bc	18.52d
	BS	1.29d	51.32a	7.56d	1.36ab	0.92a	22.34a
	BC	1.31de	50.57a	8.24a	1.24c	0.88a	19.12d

Different lowercase letters in the same column indicate significant difference among the treatments (Fisher’s least significant distance test, *P* < 0.05).

CK: control treatment, with conventional farming fertilizers; BS: biogas slurry treatment; BC: biochar treatment.

### Physical properties of the top soil

Compared with the results prior to the experiment, the bulk density of soil in 0–20 cm (Table 1) was decreased by the BC and BS treatments, especially after 2012. Compared with that of the CK, the bulk density after wheat harvest in 2013 decreased by 2.90% (*P* < 0.05) and by 6.52% with the BS and BC treatments, respectively. The total soil porosity with the BS treatment increased by 3.15%, but with the BC treatment, it significantly increased by 7.09%. The results in 2014 showed that, compared with that of the CK, the bulk density decreased by 5.15% and by 3.68% with the BS and BC treatments, respectively, with no significant difference between both the treatments (*P* < 0.05).

**Figure 1:**
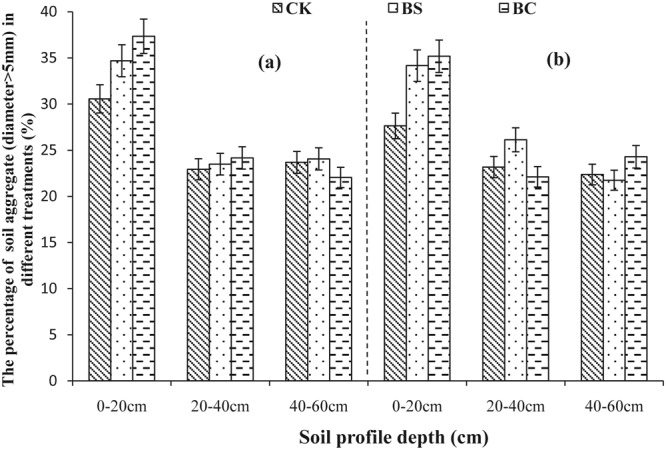
Percent of water-stable aggregate (diameter >5 mm) with different treatments (CK: conventional fertilizers; BS: biogas slurry treatment; BC: biochar treatment) in 2013–2014.

**Figure 2:**
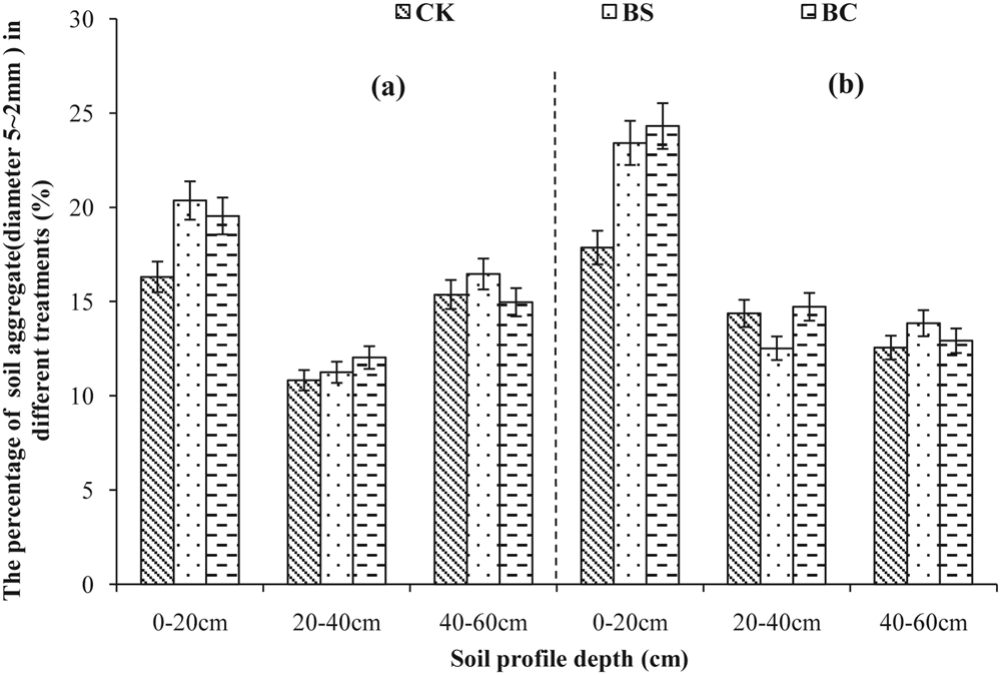
Percent of water-stable aggregate (diameter 5–2 mm) with different treatments (CK: conventional fertilizers; BS: biogas slurry treatment; BC: biochar treatment) in 2013–2014.

**Figure 3:**
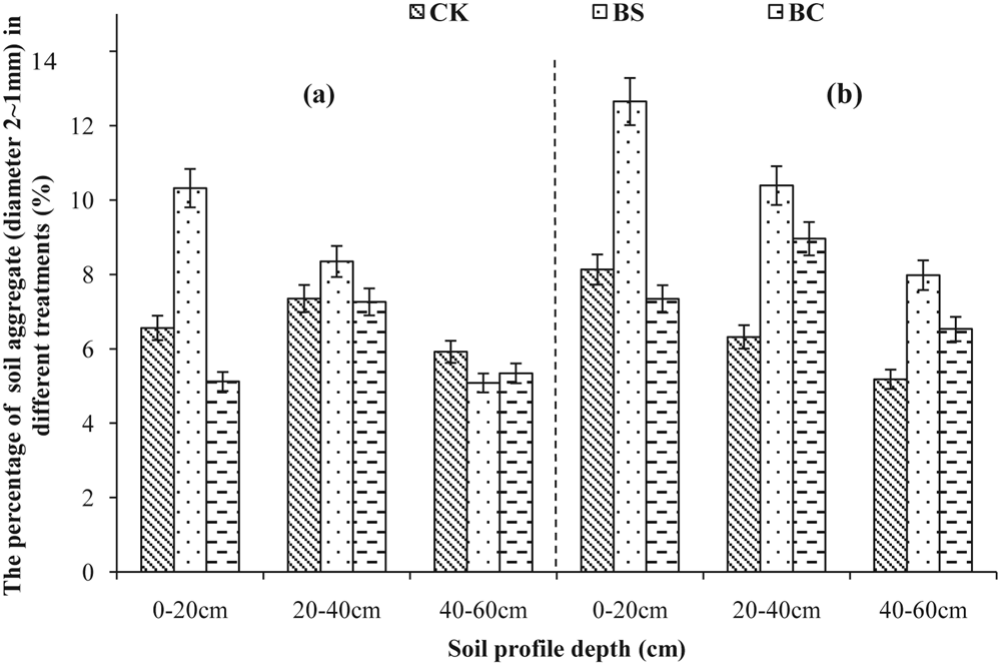
Percentage of water-stable aggregate (diameter 2–1 mm) with different treatments (CK: conventional fertilizers; BS: biogas slurry treatment; BC: biochar treatment) in 2013–2014.

**Figure 4:**
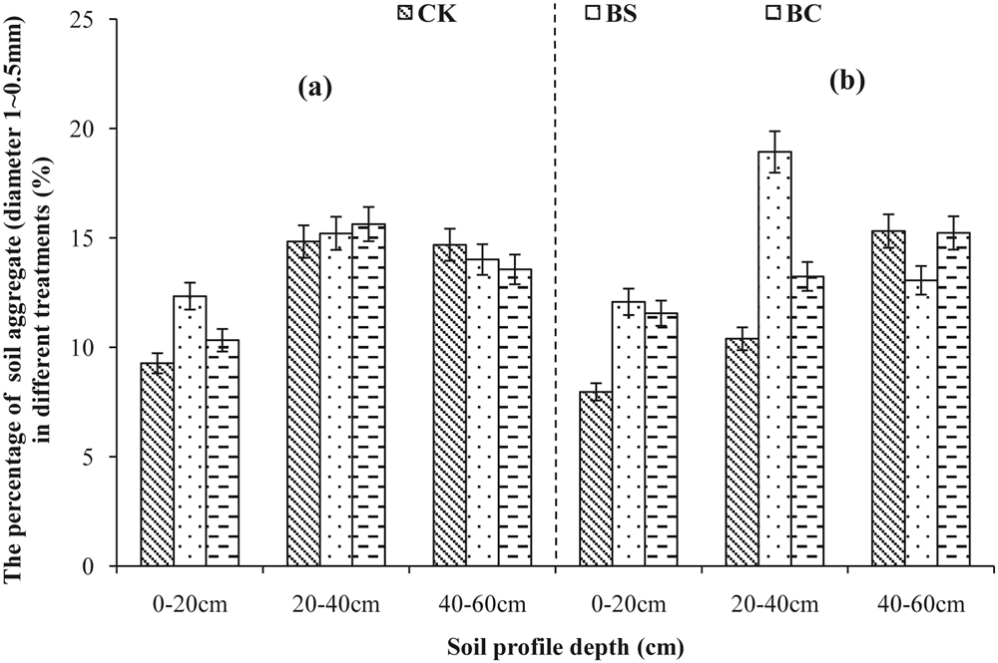
Percent of water-stable aggregate (diameter 1–0.5 mm) with different treatments (CK: conventional fertilizers; BS: biogas slurry treatment; BC: biochar treatment) in 2013–2014.

**Figure 5:**
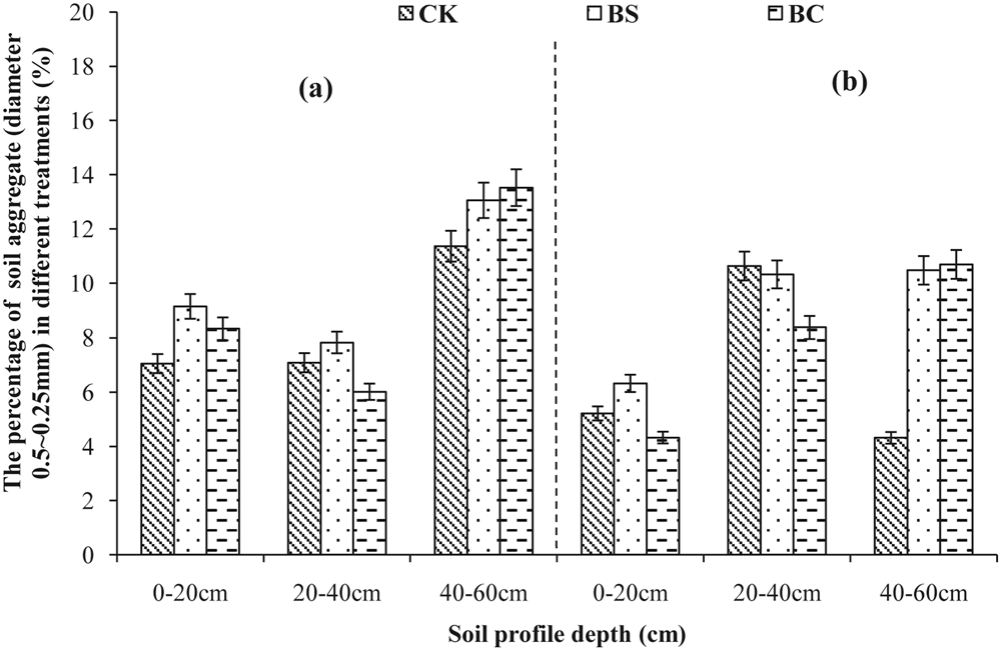
Percent of water-stable aggregate (diameter 0.5–0.25 mm) with different treatments (CK: conventional fertilizers; BS: biogas slurry treatment; BC: biochar treatment) in 2013–2014.

Aggregate size distribution in 2013 and 2014 is shown in Figs 1–5. A comparison of soil layers revealed a decrease in aggregates >5, 2–5, and 1–2 mm (Figs 1–3) along the soil profile (0–60 cm) (*P* < 0.05). The order of decrease of aggregates >5 mm in the 0–20 cm layer (Fig. 1) was BC > BS > CK (*P* < 0.05), the order decrease of 2–5 mm aggregates in the 0–20 cm layer (Fig. 2) was BS > BC > CK in 2013, while, it was BC > BS > CK in 2014 (*P* < 0.05). The order of decrease of 1–2 mm aggregates in the 0–20 cm layer (Fig. 3) was BS > CK > BC (*P* < 0.05), the order of decrease of 0.5–1 mm aggregates in the 0–20 cm layer (Fig. 4) was BS > BC > CK, and that of the 0.25–0.5 mm aggregates in the 0–20 cm layer (Fig. 5) was BS > BC > CK in 2013 (*P* < 0.05).

### Microbial quantity and enzyme activity

Table 3 shows that after four years of rotation of wheat/maize, the BS and BC treatments significantly increased the bacterial populations at rates of 258% and 121%, increased actinomycete populations at rates of 95.1% and 29.6%, respectively, in comparison with those of the CK. The number of fungi decreased by 21.9% with the BS treatment, but increased by 33.3% with the BC treatment, compared with that of the CK. Soil urease and protease activities increased by 68.72% and 28.24% with the BS treatment in comparison with those of the CK, whereas there were no significant differences in the activity of these enzymes with the BC treatment.

**Table 3:** Soil microbial abundance and enzymatic activity in the 0–20 cm soil layer with different treatments in 2014.

Treatment	Soil microbial quantity			Enzymatic activity (U/g)	
	Bacteria (10^6^)	Fungus (10^3^)	Actinomycetes (10^5^)	Urease	Protease
CK	2.04^c^ ± 0.05	1.14^b^ ± 0.06	0.81^c^ ± 0.02	1.86^b^ ± 0.07	783.25^b^ ± 73.37
BS	7.32^a^ ± 0.35	0.89^c^ ± 0.11	1.79^a^ ± 0.05	2.81^a^ ± 0.20	1002.36^a^ ± 92.41
BC	3.98^b^ ± 0.12	1.52^a^ ± 0.024	1.05^b^ ± 0.07	1.78^b^ ± 0.10	836.32^b^ ± 55.95

Different lowercase letters in the same column indicate significant difference among the treatments (Fisher’s least significant distance test, *P* < 0.05).

CK: control treatment, with conventional farming fertilizers; BS: biogas slurry treatment; BC: biochar treatment.

### Crop yield and grain quality

The results of the four-year field study (Table 4) showed that crop yields and grain quality of the CK dropped annually, except crude protein in the maize, which increased in 2014. The grain yield, grain crude protein content of wheat, and grain crude protein and grain starch content of maize with the BS treatment decreased in 2013, and then increased in 2014, whereas, an opposite tendency was observed for wheat grain starch. Crops yield with the BC treatment exhibited an overall decreasing trend during 2012–2014, whereas, the maize grain starch content with the BC treatment increased annually. Compared with those of the CK, the BS treatment improved crop yield and grain quality; and the BC treatment increased crop yield in 2013 and 2014, and improved wheat grain crude protein content in 2013, and maize grain starch content in 2012 and 2014 (*P* < 0.05).

**Table 4:** Crop yield and grain quality with different treatments in 2012–2014a.

Year	Treatment	Winter wheat			Summer maize		
		Yield (kg/hm^2^)	Grain starch (%)	Grain crude protein (%)	Yield (kg/hm^2^)	Grain starch (%)	Grain crude protein (%)
2012	CK	7989d	53.16c	17.16c	13162d	72.16d	10.24c
	BS	9023a	54.83b	18.25ab	14103a	75.01a	12.78a
	BC	7896de	50.79d	16.83cd	13025de	68.38f	11.43b
2013	CK	7595f	50.77d	15.76d	12036g	69.85e	9.68c
	BS	8647c	56.64a	17.25bc	13820b	73.69bc	11.23b
	BC	7953d	53.19c	16.03d	12965e	70.13e	10.26c
2014	CK	7205g	49.25e	16.15d	11863h	71.08e	11.56b
	BS	8896b	54.46bc	18.62a	13698b	74.32ab	13.18a
	BC	7815e	50.34de	15.68d	12596f	72.73cd	11.07b

Different lowercase letters in the same column indicate significant difference among the treatments (Fisher’s least significant distance test, *P* < 0.05).

CK: control treatment, with conventional farming fertilizers; BS: biogas slurry treatment; BC: biochar treatment.

## Discussion

The BS treatment, which is rich in polysaccharides and humic acids, reduced the soil pH and improved pH buffering. In contrast, the pH of soil with the BC treatment remained largely unchanged over the four-year experimental period. The application of BC and BS increased not only the soil TN and TP content, but also the OM content, including polysaccharides and humic acids, which facilitate soil aggregation. This result is similar to that reported by Möller *et al*.^27^. Biochar has pores of size ranging from nanometres to a few microns, resulting in a large Brunauer–Emmett–Teller (BET) surface area (78.6 m^2^/g), although the area of biochar in the present study was lower than that stipulated in the EU guidelines 2012. It still aided in adsorbing nutrients, especially those that are less mobile, such as phosphorus, increasing the total nutrient content (Table 2). In addition, biochar aids in soil aggregation and increases soil OM content^28^.

Laird *et al*.^11^ and Glaser *et al*.^29^ showed that amending soil with biochar decreased soil bulk density and increased macro-aggregate content, which is consistent with our results shown in Table 2 and Figs 1–5, because of significantly lower density (0.39 g/cm^3^) of biochar than soil bulk density (1.39 g/cm^3^, Table 1). In addition, biochar boosts microbial activity^30^ and facilitates soil aggregation as shown in Figs 1–5, thereby, improving the soil structure. Our results are in agreement with those of other studies^31,32^. However, other studies have showed that biochar does not significantly increase aggregate size, suggesting that aggregates formed early after application can be broken down due to tillage at planting and weeding^33,34^. The differential effects can also be explained by differences in time, application rate, texture of the biochar used^6^, and controlled conditions during the production of biochar. A recent study showed that open burning could also produce char and result in differing physicochemical properties, but these wildfire charcoals have lower carbon sequestration potentials than biochars^35^. Biogas slurry, especially, the residue in biogas slurry tanks, is rich in humic substances^18^ and hence can increase soil microbial activity and aggregation, improving soil structure as a result.

In the present study, biochar increased the soil porosity more than that by the biogas slurry, whereas, the biogas slurry had a more significant effect in increasing soil aggregation than that by biochar. It is noteworthy that the BC treatment increased aggregates of size >2 mm at the depth of 0–20 cm, whereas, the BS treatment increased aggregates of size 0.25–0.5 mm. This difference was likely due to the pores and specific surface area of biochar, which can adsorb and fix a variety of inorganic ions and polar or non-polar organic compounds, increase soil microbial activity^28^, and accelerate the formation of stronger aggregates producing organically and inorganically mediated large aggregates^36,37^. In contrast, the improvement in soil structure with the addition of biogas slurry was likely due to its high specific surface area, humic acid content, and chemical bonds of hydrophilic colloid^38^.

The results of the present study showed that soil bacterial, actinomycete, and fungal populations significantly increased with the BC treatment. The BS treatment significantly increased the abundance of bacteria and actinomycetes, whereas, it decreased the fungal biomass. As biochar has a substantial specific surface area and pore system, it can significantly improve gas flow in the soil, thereby, providing nutrients and habitat for microorganisms to grow^39^. On the contrary, the application of biochar and nitrogen fertilizer provides sufficient carbon and nitrogen for microbes, promoting microbial activities and growth^40^. Biogas slurry is rich in a variety of nutrients, and the addition of carbon to soil promotes the growth of soil microorganisms, while gibberellin, NH_4_^+^, vitamin B, and other matters present in the anaerobic biogas slurry suppress fungal growth^41^, thereby, reducing fungal mass.

The observed increase in the activity of urease and protease following BS application is consistent with the findings of Tang *et al*.^42^, who reported that after the application of biogas slurry, soil invertase, urease, and phosphatase activities increased. This is because slurry is rich in nutrients and bioactive substances, which enhance enzyme activity and promote changes in the soil physiological and biochemical processes. In the present study, the soil TN and OM content with the BS treatment were higher than those in the CK, but the pH of soil was lower than that in the CK. Similar to our results, Huang *et al*.^43^ reported a positive correlation between urease and protease activities and TN and OM content in soil, and a negative correlation with soil pH. The interaction between soil microorganisms and biochar is affected by various factors, such as experimental conditions in field, soil texture, soil fertility, land use, and nutrient management^44–46^. For example, the above studies have reported that the application of yeast-derived biochar to soil increases fungal mass, whereas, the application of glucose-derived biochar suppresses the growth of gram-negative bacteria.

Crop yield and grain quality depend on the availability of water and nutrients, as well as on soil structure. Extensive studies have indicated that increasing the soil OM content improves soil ventilation and aggregation, benefiting crop growth and hence its yield^47^. Manna *et al*.^48^ reported that the application of organic and/or chemical fertilizer improves soil productivity in soybean-wheat crop rotation. López-Valdez *et al*.^49^ showed that the application of organic fertilizer increases soil OM content and nutrient bioavailability, thus, increasing crop yield. Moreover, Pan *et al*.^50^ analysed the mechanism of protein and starch synthesis in wheat, and found that their accumulation is closely related to the availability of nitrogen and water, as well as to soil temperature.

A study has showed that soil moisture content and water-holding capacity with the hoggery biogas slurry and peanut-shell biochar treatments were higher than those with the CK^5^. An increase in soil moisture content improves photosynthesis, which in turn increases the grain filling rate and yield^51^. Besides, the increase in OM and TN content with the BS and BC treatments were reflected in increased crop biomass, yield, crude protein, and starch content in the grains. The increase in ventilation, porosity, and macro-aggregates with the BS and BC treatments favour root respiration and growth, thus, increasing crop yield^52^. In addition, the increase in microorganisms and enzyme activities with the BS treatment (Table 3) also enhanced the bioavailability of nutrients to the plant roots. Soil microbes play a critical role in nutrient cycling and energy flow in ecosystems, and all biogeochemical reactions in the soil are mediated by enzymes. Increased soil microorganisms and enzyme activity enhance the content of vitamins, amino acids, and organic acids in soil, which can trigger a priming effect and further enhance the bioavailability of nitrogen to plants^53,54^, consequently improving yield and grain quality. It is noteworthy that in the present study, the addition of biochar improved soil properties and crop yield. Although the benefit of crop yield cannot cover the expense of biochar (2200 yuan/t*28t), the improvement effect of biochar on soil fertility contributes for sustainable productivity and environment for a long term.

In conclusion, this four-year field experimental study on the effect of amending soils with peanut-shell biochar and biogas slurry led to the following conclusions:

- Compared with the background value and those of the CK, amending the soil with biogas slurry and biochar improved the soil TN, TP, and OM content. The biogas slurry decreased soil pH, and no significant change in pH was found in soil with the BC treatment.
- Compared with those of the background and the CK, amending the soil with biogas slurry and biochar improved soil structure and increased soil water-stable macroaggregates.
- Compared with those of the CK, both BS and BC treatments significantly increased microbial biomass and actinomycetes abundance. The addition of biochar increased fungal mass and with the addition of biogas slurry increased urease and protease activities, when compared those of the CK.
- Compared with those of the CK, both BS and BC treatments improved the yield of both winter wheat and summer maize, and biogas slurry also increased crude protein and starch content in the grains of both crops.

## Materials and Methods

### Experimental sites and soil

The field experiment was conducted in the Agricultural Water and Soil Environment Scientific Field Observation Station at the Chinese Academy of Agricultural Sciences, located in Xinxiang, Henan Province (35°19′N 113°53′E; 73.2 m altitude). The mean annual temperature is 14.1 °C, frost-free period is for 210 d, sunshine duration is 2398.8 h, mean annual precipitation is 588.8 mm, and mean annual evaporation is 2000 mm. The experiment was conducted from June 2010 to June 2014. The experimental crops were summer maize and winter wheat, in rotation (starting from 2010, maize was planted every year in June and harvested at the end of September; wheat was planted during early October and harvested during the first half of June the following year). Major soil properties prior to the experiment are listed in Table 1.

### Biochar and biogas slurry

Hoggery biogas slurry was obtained from the Xinxiang Shengda Livestock Husbandry Company (Xinxiang, China), which has a large-scale microbial anaerobic fermentation processing system. The biogas slurry used in the experiment had a pH of 6.35 and contained 650–900 mg N/L total nitrogen (TN), 3.25–11.15 mg/L total phosphorus (TP), and 639–1189 mg/L chemical oxygen demand (COD). The raw material for biochar was peanut shells, which were purchased from ShangqiuSanli New Energy (Henan, China) and were processed in a continuous vertical biomass carbonization furnace at 350 °C–500 °C. The pH of biochar was 9.12, and contained 461.78 g/kg organic carbon, 6.8 g/kg TN, 3.9 g/kg TP, 18.3 g/kg H, 130.8 g/kg O; the H/C_org_ molar ratio was 0.55 (<0.7, meets the standards of the IBI and EU guidelines 2012), O/C_org_ molar ratio was 0.25 (<0.4, meets the standards of the EU guidelines 2012), cation exchange capacity (CEC) of biochar was 33.6 mmol/kg, BET surface area of biochar was 78.6 m^2^/g (<150 m^2^/g, lower than that stipulated in the EU guidelines 2012), and the bulk density was 0.39 g/cm^3^.

### Experimental treatments and fertilization

Three treatment groups were set up in this experiment: conventional fertilizers (control, CK), biochar (BC), and hoggery biogas slurry (BS). Three replicates were performed for each treatment, with a total of nine small plots with an area of 3 × 6 m^2^. Completely randomized design was used. The irrigation time, frequency, and quantity were identical among the three treatments. The treatment for the CK group was based on local farming habits: 325 kg/ha of urea was applied as nitrogen fertilizer (150 kg/ha of pure nitrogen), of which 50% was used as a base fertilizer, while the remaining was applied as top-dressing during the jointing stage; 150 kg/ha of monopotassium phosphate (78 kg/ha P_2_O_5_, 51 kg/ha K_2_O) was applied as a one-time phosphate and potassium base fertilizer. During the first year (2011), the application of nitrogen, phosphate, and potassium base fertilizer was identical for the three treatments, and the biochar nitrogen content was also calculated as part of nitrogen base fertilizer. After broadcast, the fertilizer and biochar were mixed mechanically into the top soil (0–20 cm). The top-dressing of nitrogen was identical among these treatments as well; urea was employed as the top-dressing fertilizer in the CK and BC treatments, and biogas slurry with an equal amount of nitrogen in the BS treatment. During the second, third, and fourth year, the fertilizers applied were same as those applied during the first year in the CK and BS treatments, and the fertilizer applied in the BC treatment was same as that of the CK, because biochar (applied in June 2011, 28 t/ha, approximately 1% of the top soil mass) was not applied during the subsequent years.

### Soil sampling and analysis

Before planting summer maize in 2010, three layers of soil— 0–20, 20–40, and 40–60 cm—were randomly sampled by the five-point composite method throughout the experimental site to measure the basic physical and chemical soil properties before the start of the experiment (Table 1). Herein, the experimental data provided are from 2011–2014. Relevant soil indicators, such as major physical and chemical indicators, in different soil layers were measured in each plot.

The soil bulk density was measured by the ring sampling (98.17 cm^3^, D = 5 cm, H = 5 cm) method. Soil water content was measured using a manometer and by oven drying. Soil texture was determined by the pipette method (International System of Units standards)^5^. Soil pH was measured using a pH meter/potentiometer (PHBJ-260 portable pH meter, 0.01 pH resolution; Shanghai Precision & Scientific Instrument, Shanghai, China). The soil OM content was measured by the potassium dichromate external heating method. The content of TN and TP was determined by continuous flow analysis (AutoAnalyzer 3, sensitivity 0.001 AUFS; Bran & Luebbe, GmbH, Norderstedt, Germany). The macro-aggregate structure was analysed by wet screening. Soil microbial properties were determined by plate count and Biolog Eco micro-plate; the abundance of bacteria, actinomycetes, and fungi were measured using Beef extract peptone medium, Gause’s synthetic agar medium, and Martin’s medium, respectively^55^. Soil urease activity was determined by the phenol-sodium hypochlorite colorimetric method and protease activity was determined by ninhydrincolorimetry. The average crop yield was measured by harvesting five representative rows of plants from each plot that were air-dried and weighed. The crude protein and starch content in grains were determined by Kjeldahl’s method and the anthrone colorimetric method, respectively^55^.

### Statistical analyses

All data were analysed using the SPSS software (IBM corporation, Armonk, NY, USA) and the results are expressed as the arithmetic mean value ± standard deviation. The differences in the means were compared by the least significant difference (LSD) test at P < 0.05.
